# Characterization of the haemagglutinin properties of the H5N1 avian influenza virus that caused human infections in Cambodia

**DOI:** 10.1080/22221751.2023.2244091

**Published:** 2023-08-25

**Authors:** Pengxiang Chang, Jiayun Yang, Thusitha K. Karunarathna, Mehnaz Qureshi, Jean-Remy Sadeyen, Munir Iqbal

**Affiliations:** a The Pirbright Institute, Pirbright, Woking, GU24 0NF, UK; b Royal Veterinary College, Hawkshead Lane North Mymms, Hertfordshire, AL9 7TA, UK; c Institute of Infection, Veterinary and Ecological Sciences, University of Liverpool, Liverpool L3 3RF, UK

**Keywords:** High pathogenicity avian influenza (HPAI) H5N1, zoonotic fatal Infections, Cambodia, receptor binding, fusion pH, haemagglutinin thermal stability, antigenicity, poultry

## Abstract

High pathogenicity avian influenza (HPAI) H5N1 is a subtype of the influenza A virus primarily found in birds. The subtype emerged in China in 1996 and has spread globally, causing significant morbidity and mortality in birds and humans. In Cambodia, a lethal case was reported in February 2023 involving an 11-year-old girl, marking the first human HPAI H5N1 infection in the country since 2014. This research examined the zoonotic potential of the human H5N1 isolate, A/Cambodia/NPH230032/2023 (KHM/23), by assessing its receptor binding, fusion pH, HA thermal stability, and antigenicity. Results showed that KHM/23 exhibits similar receptor binding and antigenicity as the early clade 2.3.2.1c HPAI H5N1 strain, and it does not bind to human-like receptors. Despite showing limited zoonotic risk, the increased thermal stability and reduced pH of fusion in KHM/23 indicate a potential threat to poultry, emphasizing the need for vigilant monitoring.

High pathogenicity avian influenza (HPAI) H5N1 virus is a subtype of the influenza A virus that primarily infects birds. A novel genotype of HPAI H5N1 arose in China in 1996 and has since spread to other countries in Asia, Europe, and Africa [1]. HPAI H5N1 viruses are highly transmissible and lethal in avian species, especially in gallinaceous domestic poultry. HPAI H5N1 virus can also infect humans occasionally. To date, there have been 868 human cases of H5N1 infections globally, 457 of which died from infection with a mortality rate of approximately 53% [2]. Human infection occurs via proximity contact with infected birds, virus-contaminated surfaces, or respiratory secretions. Family clustering of the HPAI H5N1 virus has been reported and raised concerns about human-to-human transmission and thus possible pandemic potential [3].

On 23 February 2023, a lethal H5N1 human infection case was reported in Prey Veng province, southern Cambodia. The victim was identified as an 11-year-old girl [4]. The father of the index case was also infected and remained asymptomatic. All 11 close contacts tested negative for H5N1. Sequence analysis shows the H5N1 virus belongs to clade 2.3.2.1c, which has been circulating in poultry in southeast Asia since 2014 [4]. These are the first two human cases of HPAI H5N1 infections reported from Cambodia since 2014. From 2003 to 25 February 2023, there have been a total of 114 human infections of the H5N1 virus reported in Cambodia, and 75 cases were found to be lethal [4].The haemagglutinin (HA) of the avian influenza virus (AIV) plays a key role in the interspecies transmission. The shift from avian to human receptor binding preferences, along with increased acid and thermal stability of HA, has been closely associated with human pandemic potential of avian influenza viruses [5]. This association is further supported by the transmission study of H5N1 AIVs using the ferret model [6,7]. To investigate the zoonotic risk of the human H5N1 isolate, we generated the recombinant viruses via reverse genetics (RG) containing HA and neuraminidase (NA) from the clade 2.3.2.1c HPAI H5N1 viruses and the six internal segments from laboratory adapted A/Puerto Rico/8/34 (H1N1) (PR8) virus. We investigated the risk of the recent H5N1 AIV (A/Cambodia/NPH230032/2023, accession numbers EPI2419700 and EPI2419702, referred to as KHM/23) to human and animal health by assessing the virus receptor binding, pH of fusion, HA thermal stability, and the antigenicity change with reference to earlier clade 2.3.2.1c HPAI H5N1 virus (A/duck/Vietnam/OIE-2202/2012, referred to as VNM/12 [8]).

The receptor binding specificity of influenza A virus is one of the major determinants of host specificity. It is a prerequisite for pandemic viruses to switch receptor binding specificity from avian receptor Siaα2-3Gal to human receptor Siaα2-6Gal [9]. To examine the receptor binding of the H5N1 AIVs that cause human infections in Cambodia, we utilized biolayer interferometry to characterize the receptor-binding profiles of KHM/23 H5N1 to the avian-like 3′-sialylacetyllactosamine (3SLN) and human-like 6′-sialylacetyllactosamine (6SLN) receptor analogues. In agreement with previous reports [10], the H1N1 (A/California/7/2009, referred as pdm/09) and H5N1 VNM/12 as control viruses only bound to 6SLN or 3SLN respectively (Figure 1A and B). Similar to VNM/12, the human isolate KHM/23 did not show any detectable binding towards human-like 6SLN receptor analogues (Figure 1C). It is worth noting that the KHM/23 showed relatively weaker binding to 3SLN receptor analogues when compared with the VNM/12.

**Figure 1:**
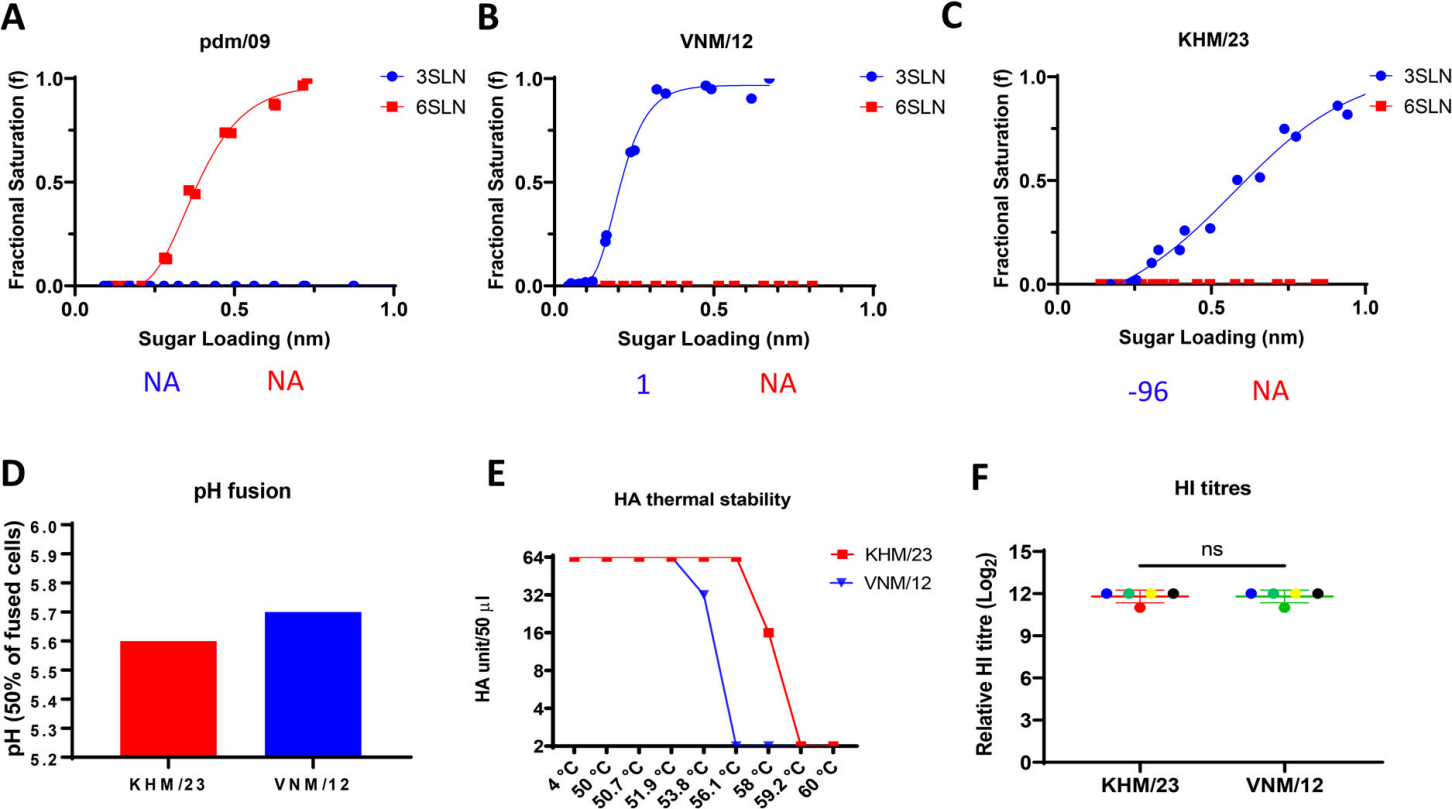
. Receptor binding, pH of fusion, thermal stability, and antigenicity characterization of the H5N1 avian influenza virus that caused human infections in Cambodia. The binding of purified reassortants (A) H1N1 pdm/09 (A/California/7/2009), (B) H5N1 VNM/12 (A/duck/Vietnam/OIE-2202/2012), and (C) H5N1 KHM/23 (A/Cambodia/NPH230032/2023) to avian and human receptor analogues were measured by biolayer interferometry. The numbers below each figure show the fold change of receptor binding of indicated viruses to avian (α−2,3-SLN, shown in blue) and human (α-2,6-SLN, shown in red) receptor analogues compared to those of the H5N1 VNM/12. “−”: reduction; “NA” indicates not applicable. Data is the combination of two repeats for each virus and receptor analogue combination. (D) The pH fusion of H5N1 KHM/23 and VNM/12. Syncytium formation in Vero cells infected with reassortant H5N1 KHM/23 and VNM/12 and the pH, at which 50% of maximum syncytium formation was taken as the predicted pH of fusion. (E) HA thermal stability of reassortant H5N1 KHM/23 and VNM/12. 64 HA units of reassortant virus were either left at 4.0 °C as control or heated at 50 °C, 50.7 °C, 51.9 °C, 53.8 °C, 56.1 °C, 58.0 °C, 59.2 °C and 60 °C for 30 min before the HA assay. (F) the antigenicity characterization of KHM/23 by Hemagglutination inhibition (HI). HI assay with four individual and single pooled chicken polyclonal post-vaccinated antisera raised against H5N1 VNM/12. “ns” indicates not significant (*P* > 0.05). The reassortant H5N1 AIVs and the human H1N1 were generated by reverse genetics with HA and NA from indicated H5N1 AIVs or human H1N1 and the internal segments from PR8 H1N1 virus.

The pH of fusion has been shown to play an important role in host adaptation and transmission; human-adapted influenza viruses generally fuse at a lower pH (5.0 to 5.4), while AIVs normally have a higher pH fusion threshold (5.6 to 6.2) [5]. Consistent with the previous report, VNM/12 triggered fusion at pH 5.7 [10]. Similar with VNM/12, it appears that KHM/23 fused at a slightly lower pH 5.6 (Figure 1D). In addition to receptor binding and pH of fusion, increased HA thermal stability has been linked to a vital role in AIV evolution [11]. Previous work has shown that the HA of aerosol or respiratory droplet-transmissible influenza viruses are comparatively more thermal stable [6,7]. To assess the heat stability of the HA protein, the reassorted KHM/23 and VNM/12 viruses were subjected to incubation at various temperatures (4.0 °C, 50 °C, 50.7 °C, 51.9 °C, 53.8 °C, 56.1 °C, 58.0 °C, 59.2 °C and 60 °C) for 30 min, and the loss of HA activity was then determined. KHM/23 was observed to be more thermally stable in comparison to early clade 2.3.2.1c VNM/12 (Figure 1E). The HA activity of VNM/12 was undetectable at 56.1 °C, while KHM/23 lost activity at 59.2 °C. Haemagglutination inhibition (HI) assay was adopted to assess the antigenic change between KHM/23 and VHM/12 by using chicken antisera raised against inactivated VNM/12. Both KHM/23 and VHM/12 showed no difference in HI titres, suggesting that no significant antigenic drift between KHM/23 H5N1 with early clade 2.3.2.1c HPAI H5N1 virus (Figure 1F).

We comprehensively assessed the HA of human H5N1 virus isolated in Cambodia. Our results suggest that the human KHM/23 H5N1 virus exhibited similar receptor binding and antigenicity profile with the early clade 2.3.2.1c HPAI H5N1 strain. Most importantly, the Cambodia H5N1 AIV showed no detectable binding to human-like receptors. The human isolate KHM/23 was relatively more acid and thermal stable in comparison to VNM/12. Previous study indicates that H5N1 AIV with reduced pH of fusion can increase the virus transmissibility in ducks, possibly due to higher virus shedding and greater environmental persistence [12]. Reduced pH of fusion was also found to be critical for airborne transmissibility of AIV H9N2 between chickens [13]. Taken together, the HA of human H5N1 isolate from Cambodia poses limited zoonotic risk. However, the reduced pH of fusion and increased thermostability of KHM/23 may pose a potentially heightened threat for poultry, which requires close monitoring.

## Supplementary Material

Supplemental Material

Supplemental Material
