# Supplementary material for: Characterization of the haemagglutinin properties of the H5N1 avian influenza virus that caused human infections in Cambodia
Source: Emerg Microbes Infect. 2023 Aug 25;12(2):2244091. doi: 10.1080/22221751.2023.2244091 (PMC10461499; doi:10.1080/22221751.2023.2244091)
Supplement: Supplemental Material [file TEMI_A_2244091_SM2666.docx]

**SUPPLEMENT MATERIALS AND METHODS**

### Ethics Statement

### The use of embryonated eggs in this study strictly followed the guidance and regulations of the United Kingdom Home Office under project licence number P68D44CF4. The study was subject to ethical review and approval by the animal welfare ethical review board at the Pirbright Institute. All influenza virus-related work was conducted under biosafety level-2 conditions.

### Viruses and cells

The reassortant H5N1 viruses containing HA and NA from HPAI H5N1 virus (A/Cambodia/NPH230032/2023) (referred to as KHM/23), or HA from H5N1 (A/duck/Vietnam/OIE-2202/2012) and NA from H5N1 (A/turkey/Turkey/1/2005) (referred to as VNM/12). The DNA sequences for the HA and NA of the H5N1 and human pandemic H1N1 (A/California/7/2009, referred as pdm/09) viruses are derived from the Global Initiative on Sharing All Influenza Data (GISAID). The GISAID accession numbers for the HA of CAM/23, VNM/12, pdm/09 are EPI1857209, EPI408415 and EPI1593569 respectively. The GISAID accession numbers for the NA of CAM/23, VNM/12 and pdm/09 are EPI2419702, EPI118777 (A/turkey/Turkey/1/2005), EPI1593568, respectively. The HA and NA genes were synthesized by GeneArt (Thermo Fisher Scientific) and cloned into PHW2000 plasmids. The polybasic amino acid motif of the HPAI H5N1 HAs was removed. The internal gene segments from PR8 H1N1 virus (A/Puerto Rico/8/34) were generated using previously described methods [1]. The resulting viruses were propagated in 10-day-old embryonated chicken eggs, and virus stocks were stored at -80°C.

MDCK cells, human embryonic kidney 293T cells, and Vero cells (ATCC) were cultured in DMEM (Gibco) supplemented with 100 U/mL penicillin, 100 μg/mL streptomycin (Gibco), and 10% FCS (Gibco) at 37°C with 5% CO2.

**HA assay**

To determine the HA titre of the H5N1 AIVs, we followed the protocol described in the WHO animal influenza training manual [2]. In brief, 50 μl of the virus samples were serially diluted two-fold in 96-well V-bottom microtitre plates (Greiner). Next, 50 μl of 1% washed chicken red blood cells were added to each well, and the plates were incubated for 30 min at room temperature. The HA titre was then determined as the reciprocal dilution of the last well that showed no agglutination of the red blood cells and was expressed as HA units/50 μl.

**Hemagglutinin inhibition (HI) assay**

### In the experiment, 25 μl of chicken antisera raised against inactivated VNM/12 were subjected to two-fold serial dilution in 96-well V-bottom plates (Greiner) with PBS. The H5N1 KHM/23 or VNM/12 (4 HA units) was then added in equal volume to each well. After incubating the antibody-virus mixture for 1 h at room temperature, 50 μl of 1% chicken RBC was added to each well, and the plates were incubated for another 30 min at room temperature. The HI titer was determined as the log2 of the reciprocal of the dilution.

### HA thermal stability assay

### The reassortant H5N1 AIVs were diluted with allantoic fluid harvested from non-infected embryonated chicken eggs to a final concentration of 64 HA units/50 μl. To test the effect of heat on the virus, the samples were heated at various temperatures including 50°C, 50.7°C, 51.9°C, 53.8°C, 56.1°C, 58.0°C, 59.2°C, and 60°C using a PCR thermal cycler (Biorad) for 30 minutes. A control sample was also kept at 4°C. After the heat treatment, HA assays were performed to assess any changes in the viral activity.

### Syncytium formation assays

### To determine the pH of fusion for H5N1 AIV, syncytium formation assays were conducted as previously described [3]. Vero cells in a 96-well plate were infected with H5N1 AIVs, which were diluted two-fold serially in DMEM for 1 hour. After removing the inoculum, the cells were washed with PBS and DMEM medium with 10% FCS was added for 15 hours. The cells were then fixed with methanol and acetone (1:1 in volume) mixture and immuno-stained with an anti-nucleoprotein (NP) mouse mAb followed by horseradish peroxidase-labelled rabbit anti-mouse immunoglobulins (DAKO) as described previously [4]. The virus concentration with the highest dilution that still infected 100% of the Vero cells was used to infect Vero cells in a 96-well plate for 1 hour. After removing the inoculum and washing with PBS, DMEM medium with 10% FCS was added for 15 hours. At 16 hours post-infection, cells were treated with 3 μg/ml TPCK-treated trypsin for 15 minutes and then exposed to PBS buffers with pH values ranging from 5.2 to 5.9 (at 0.1 unit increments) for 5 minutes. The PBS buffer was then replaced with DMEM with 10% FCS, and the plates were further incubated at 37 °C for 3 hours to allow for syncytium formation. To visualize the syncytium formation, the cells were fixed with methanol and acetone (1:1 in volume) mixture and stained with Giemsa stain (Sigma-Aldrich) for 3 hours at room temperature. The pH at which around 50% of the maximum syncytium formation was estimated to occur was taken as the predicted pH of fusion.

### Virus purification and biolayer interferometry

### H5N1 AIVs were purified as previously described [5]. Briefly, virus harvested from allantoic fluid of embryonated eggs was pelleted by ultracentrifugation and then purified through a continuous 30 to 60% (weight/volume) sucrose gradient and resuspended in PBS buffer. The biotinylated α2,3- and α2,6-linked sialyl lactosamine sugars (3SLN and 6SLN, respectively) were purchased from GlycoNZ. Virus was diluted in HBS-EP buffer (TEKnova) containing 10 μM oseltamivir carboxylate (Roche) and 10 μM zanamivir (GSK) to a concentration of 100 pM, and the binding to receptor analogues was measured on an Octet RED instrument (ForteBio). The equilibrium responses for virus binding were plotted as a function of the amount of sugar immobilized on the biosensor calculated from the response during the sugar loading step [6]. The relative dissociation constant, as a measure of binding to 3SLN, and 6SLN was calculated.

### Statistical analysis

### Statistical analyses were performed using GraphPad Prism 8 (GraphPad Software). paired Student’s t-test was used to analyse the differences between different groups (Figure 1F). p values < 0.05 were considered significant.

**REFERENCES**

1. Chang P, Sealy JE, Sadeyen JR, et al. Amino acid residue 217 in the hemagglutinin glycoprotein is a key mediator of avian influenza H7N9 virus antigenicity. J Virol. 2018 Oct 3.

2. WHO. World Health Organization . WHO manual on animal influenza diagnosis and surveillance. [cited 2023 Apr 13]. Available from: <https://apps.who.int/iris/bitstream/handle/10665/68026/WHO_CDS_CSR_NCS_2002.5.pdf?sequence> = 1&isAllowed = y.

3. Chang P, Sealy JE, Sadeyen JR, et al. Immune Escape Adaptive Mutations in the H7N9 Avian Influenza Hemagglutinin Protein Increase Virus Replication Fitness and Decrease Pandemic Potential. J Virol. 2020 Sep 15;94(19).

4. Chang P, Yao Y, Tang N, et al. The Application of NHEJ-CRISPR/Cas9 and Cre-Lox System in the Generation of Bivalent Duck Enteritis Virus Vaccine against Avian Influenza Virus. Viruses. 2018 Feb 13;10(2).

5. Peacock TP, Benton DJ, James J, et al. Immune Escape Variants of H9N2 Influenza Viruses Containing Deletions at the Hemagglutinin Receptor Binding Site Retain Fitness In Vivo and Display Enhanced Zoonotic Characteristics. J Virol. 2017 Jul 15;91(14).

6. Lin YP, Xiong X, Wharton SA, et al. Evolution of the receptor binding properties of the influenza A(H3N2) hemagglutinin. Proc Natl Acad Sci U S A. 2012 Dec 26;109(52):21474-9.
